# General orders for the embedded researcher: Moorings for a developing profession

**DOI:** 10.1002/lrh2.10254

**Published:** 2020-12-22

**Authors:** Matthew F. Hudson

**Affiliations:** ^1^ Prisma Health Cancer Institute Greenville South Carolina USA

**Keywords:** embedded research, evidence‐based practice, learning health system, team science

## Abstract

Learning health systems increasingly welcome embedded researchers as stakeholders poised to inform evidence‐based practice. While care systems are potentially familiar with the embedded researcher tools and techniques, care systems may less frequently consider embedded research as a vocation. This insensitivity potentially reduces embedded researchers merely to instruments, as opposed to professional partners in transdisciplinary research. This discussion outlines “general orders” for embedded researchers. The general orders outline embedded researchers' fundamental identity and guide conduct as a means to encourage a shared identity among embedded researchers and clarify embedded researchers' roles in learning health system teams. Students and embedded researchers newly engaging learning health systems may particularly benefit from this rudimentary order list.

Learning health systems^1^ increasingly embrace embedded researchers^2^, ^3^ as stakeholders positioned to advance evidence‐based practice. Underscoring this increased interest, Canada invested in an infrastructure to create and support Learning Health Systems,^4^ and train health service and policy doctoral candidates and research fellows to assume an embedded research charge.^5^ Canada's program trains a cadre of aspiring embedded researchers in a number of care system‐centric competencies including program evaluation, critical thinking, knowledge translation, project management, and understanding health policy that shapes system practice.^6^ These competencies align with Forrest and colleagues' expectation that embedded researchers ask research questions relevant to health care organizations, employ and adapt research models and frameworks to health systems, and employ system science to understand how health system finance and operation may inform research and implementation.^7^ Embedded researchers mastering such competencies may more assuredly inform and advance evidence‐based practice.

While important, competencies do not define embedded researchers, nor explain embedded research as a vocation. Competencies merely demonstrate what embedded researchers “do,” not who embedded researchers “are.” Myopically defining embedded researchers by their competencies is as disadvantageous as defining clinical practitioners merely by their clinical tasks.^8^, ^9^, ^10^, ^11^ Therefore, the field of embedded research should make strides to clarify the vocation. Framing embedded research as a vocation may better position those embedded to advance transdisciplinary scholarship indicative of team science.^12^ Specifically, transdisciplinary scholarship requires different fields collaborating over extended periods to develop shared conceptual and methodological frameworks that transcend stakeholders' respective disciplinary perspectives.^12^ Embedded researchers can best steward transdisciplinary scholarship when they “know thyself”^13^—both for its own sake, and to better understand other stakeholders. How then might embedded researchers discover, or recall, their identity? Embedded researchers may begin by considering clinician identity^14^ and culture^15^ and healthcare organizational identity^16^ and culture^17^ (and potential clinician/organizational conflict portending dysfunction, such as workforce burnout^18^). Likewise, embedded researchers may reference academicians' identity and culture^19^, ^20^, ^21^, ^22^ informing conduct codes in academic settings.^23^ However, the collective discourse less readily articulates identity, culture, and conduct code axiom(s) for the embedded researcher‐one charged to negotiate dual affiliation^3^ (academic and clinical) within healthcare organizations. It is advantageous, then, to posit culture and identity‐informed codes to guide embedded researchers in conducting their duties. Codes may clarify embedded research expectations, both for the researcher and their differently trained colleagues.

These codes may act as a compass particularly benefitting graduate students, early career researchers, and senior scholars less facile in the clinical environment. General orders may reveal the tacit assumptions that should align practice behaviors with espoused values.^18^ Also, embedded researcher general orders may more deeply educate clinically trained colleagues on culture, practice, and identity embedded researchers' bring to the clinical enterprise. This later opportunity potentially fertilizes transdisciplinary research collaboration essential to advancing evidence‐based practice. The discourse below provides an embedded researcher code of ethics.

**Embedded Researchers will**: Assume responsibility for generating, synthesizing, and imparting clinical evidence on behalf of the patients and communities embedded researchers serve. Clinical practitioners assume primary responsibility for providing a clinical diagnosis for a patient. Likewise, embedded researchers should assume primary responsibility for asserting the scientific method to reveal organization and care delivery challenges and opportunities. The social context of physician power^24^ and responsibility^25^, ^26^, ^27^ challenges embedded researchers to assert and negotiate their prime responsibility for the scientific method (prime responsibility presumed by virtue of embedded researchers' degree and training). Embedded Researchers should trust that their training justifies their contribution to hypothesis development and leadership in study design.

**Embedded Researchers will:** *conduct oneself as an educated/learned person, vigilant to address the unknown, unproven, or misguided*. This order obliges embedded researchers to engage and inform the scientific method, and willingly explore potential bias informing hypothesis testing.^28^ Moreover, this general order obliges embedded researchers to fundamentally consider/challenge “who” determines the research topic, and the purpose the research serves. This latter obligation is particularly important given many learning health aspire to advance patient‐centered outcomes research informed by patients' values and preferences.^29^

**Embedded Researchers will:** *report all deviations from the truth to which evidence obliges a testimony*. Embedded researchers should steward efforts to report all findings, accurately and comprehensively. Embedded researchers should guard against selective reporting of an entire study (eg, failing to report all analyzed outcomes), selective reporting of specific outcomes (eg, selected follow up intervals), and incomplete reporting of specific outcomes (eg, failing to report nonsignificant *P* values).^30^, ^31^ Embedded researchers are also responsible for duly considering potential bias in selective comparative efficacy and effectiveness assessments.^32^ Transparent reporting poses embedded researchers a particular challenge when such data may reflect poorly on (or prove costly for) the care system they represent.^33^

**Embedded Researchers will:** *disseminate evidence beyond my own community, to all institutions, and communities*. As embedded researchers should evidence transparency within their health care confines (see above), they are also bound to broadly disseminate among care practitioners.^34^, ^35^, ^36^ More important, embedded researchers should steward dissemination among patients and communities,^37^ the constituents health care serves. Embedded scholars should explicitly consider dissemination in historically disadvantaged communities,^38^ as one mean to blunt persisting disparities.^39^, ^40^

**Embedded Researchers will:** *appropriately discern when lacking expertise requires deference to a more or differently experienced colleague*. To advance team science, it is necessary for the embedded researcher to discern the limits of their subject matter knowledge, or the context undergirding the problem in question. Embedded researchers may fail to seek counsel owing to imposter syndrome‐motivated fear their ignorance proves them a fraud.^41^ Clinicians and health administrators (as well as embedded researchers) should particularly attend to this potential order violation. Embedded researchers are typically gravely outnumbered in the clinical enterprise. Embedded researchers may not have the luxury of consulting with a similarly trained colleague to address a problem. Less developed care systems may lack a critical mass of embedded researchers that can convene to consider cases in a manner akin to a tumor board or grand rounds. The clinical enterprise may overly assume or hold unrealistic expectations regarding an embedded scholars' expertise depth and breadth. No clinical enterprise expects a single Medical Doctor to exercise command over the vast array of biomedical challenges that befall the human condition. Likewise, care systems should not expect a single Ph.D. or Dr.P.H. to exercise command over the myriad of epistemological or methodological challenges facing patients, care systems, or communities. Appropriate and transparent discussion regarding knowledge depth and breadth may better ensure appropriate problem address, and clarify where care systems require additional guidance.

**Embedded Researchers will:** *be vigilant for mentorship opportunities*. The clinical enterprise is amenable to research mentorship.^42^ The embedded researcher should attend to capitalizing on these mentorship opportunities—to both learn and teach. Peer‐assisted learning models^43^ can be adapted to teach care teams to consider pragmatic designs,^44^ and other embedded research competencies.^7^

**Embedded Researchers will:** *eschew bawdy, gratuitous, provocative, or demeaning commentary/behavior*. Clinical practitioners ascribe to a code of professional conduct,^45^ as do public health practitioners^46^ and other disciplines that birth embedded researchers. However, codes themselves do not protect against sexual harassment^47^ and racial bias^48^, ^49^ unfortunately perpetrated by some colleagues and staff. Embedded researchers, as evolving specialists, should particularly comport themselves discretely. Apart from their inherent immorality, frivolous or lewd discourse or acts erode team member confidence in the perpetrator's capacity to self‐regulate. Disparaging remarks or conduct also raise doubt that the embedded researcher can justly execute their charge. This confidence crisis may quickly morph into doubting or forgoing the researcher's expertise. More important, patients privy to ill comportment may certainly lose trust in care teams and the organization. Embedded researchers should remember they labor in a patient service enterprise with their clients acutely aware of the stakes embedded clinical practice. Embedded researchers should also diagnose and challenge systemic and institutional racism^50^, ^51^ portending both patient and workforce inequities.

**Embedded Researchers will:** *employ formal salutation when engaging colleagues (superiors, peers, and subordinates) in professional settings*. Professional comportment includes conveying respect for team members' mutual commitment to advancing patient and community welfare. Embedded researchers should convey this by employing formal salutation to all colleagues in the clinical setting. Greeting colleagues as “Mr.,” “Ms.,” “Dr.,” “Reverend,” and so on (unless the colleague requests otherwise) both show respect and may subtly remind colleagues they hold a lofty responsibility to advance evidence‐based practice. Ensuring respectful salutation to all team members (regardless of senior or junior standing) confers respect for their contribution to the enterprise.

## 1 DISCUSSION

How might the embedded researcher apply for these general orders? The discussion below will entertain individual and teams, organizations, and international considerations for general order application.

### 1.1 Individual and teams

First, these general orders may inform an embedded researcher's mission statement‐core/personal belief(s) motivating the embedded researcher to assume this vocation.^52^ Such a mission statement may help an embedded researcher consider their alignment with a learning health system's social, built, or administrative environment. Health system/researcher alignment, per these general orders (and, by extension, an embedded researcher's individual mission statement), may help embedded researchers fundamentally determine whether their current environment is amenable to their charge. Additionally, these general orders may encourage reflection begetting self‐awareness of developing abilities, skills, values, and interests. This self‐awareness may inform the researcher's identity, and guide the researcher toward continued professional development.^53^, ^54^ To the latter, Warden^55^ observes professional development (eg, self‐directed learning, systems change, and quality improvement) not only teaches people how to apply solutions but focus on actual performance and how to identify problems. Continuing professional development extends beyond content knowledge mastery, and encourages personal, communication, managerial, and team‐building skills optimizing reflection and problem identification.^55^ Thus, these general orders informs an embedded researcher's personal mission statement begetting continuing professional development that ultimately encourages clinical challenge identification and address.

Second, these general orders may enhance health care team function, a significant contributor to health care quality and safety.^56^ Mitchell and colleagues^57^ note five principles of team‐based health care: shared goals (clearly articulated, understood, and supported by all team members), clear roles (establishing expectations for each team members' functions, responsibilities, and accountabilities), mutual trust (creating strong norms of reciprocity and shared achievement), effective communication (consistent channels for candid and complete communication), and measurable processes and outcomes (implementing reliable and timely feedback on goal successes and failures used to track and improve performance). The general orders described above may encourage embedded researchers and their teams to articulate shared goals, clarify roles, and communicate effectively. Embedded researchers articulating (and behaving in accordance with) these general orders may more seamlessly advance trust within health care teams, and implement feedback advancing care innovation.

### 1.2 Organizational context

How may the proposed general orders align with a learning health system's pre‐existing values? Broadly, Menar and colleagues^58^ observe learning health system values include adaptability, equity, inclusiveness, open innovation, scientific integrity, shared accountability, solidarity, and transparency. Harrison and Taylor's^59^ earlier work similarly observed stewardship, care quality and access, organizational excellence, decency, and fairness among other values care‐providing organizations espouse. These altruistic values align well with the general orders described here. More pointedly, Faden and colleagues^60^ propose an ethics framework for learning health systems. This framework includes seven “obligations” for a learning health system, among these is respecting the clinical judgment of physicians. Faden et al further observe that multiple influences shape clinician assessments regarding how to best care for a patient‐notably, personal professional experience, colleague and mentor experience, scientific evidence, and clinician understanding of patients' values and preferences. Here, embedded researchers ascribing to the proposed general orders may provide scientific evidence optimally informing clinical judgment. Further, the proposed general orders may prepare embedded researchers to ensure clinicians appropriately (or equally) weigh numerous judgment influencers, as opposed to over‐reliance on one (eg, colleague and mentor experience). That is, embedded researchers operationalizing these general orders may both tacitly and explicitly encourage clinicians to duly consider and integrate all potential influences on care decisions—a particularly important contribution given Faden and colleagues' concession “even the most well‐intentioned judgements of clinicians can be subject to some form of bias (p.s21).”

Riser^61^ cautions that health care organizations potentially exhibit “institutional dissonance” evidenced by the misalignment between espoused values and actions/operations. For example, care organizations may espouse equal access for poor and disenfranchised, yet build facilities for/catering to affluent patients. This example exacerbates health inequity by both challenging access for traditionally disenfranchised populations, and disproportionately benefiting patients already socially and economically advantaged. Faden and colleagues note addressing health inequity as a learning health system's particular obligation. Faden et al admonish learning health systems to determine where systems perpetuate or exacerbate unjust inequalities, and whether/how to (re)structure learning health systems toward reducing or eliminating care inequity and discrimination. The general orders described above may encourage the embedded researcher (and their team) to alert host systems to infrastructure or activity misalignment with learning health system ethics and values; embedded research informed by these general orders may direct learning activities toward aggressive efforts to reduce or eliminate unfair or unacceptable inequalities in the evidence base available for clinical decision‐making.^60^ They may do so both to advance learning health systems' altruistic values, and comparatively more pragmatic care system values such as care quality, equity, efficiency, and sustainability.^62^

### 1.3 International context for general orders application

Embedded researchers and learning health systems are not the exclusive concern of western countries. Ghaffar and colleagues^63^ argue there is a global need for embedded research to strengthen health systems, particularly since health systems research and implementation research^64^, ^65^, ^66^ remains underfunded, globally. Given this international imperative, stakeholders may consider a country's underlying cultural values that potentially inform learning health system characteristics, and subsequent embedded researcher opportunities and challenges. Graber and Kilpatrick^67^ observed the United States health care organizations value selfless service, caring, compassion, universal care access, and individualism. Many of these values are likely evident among other countries. However, countries may differentially weigh existing values. Canada, for example, may prioritize “shared accountability”^58^ and population wellness^68^ above individualism. General order application, then, maybe calibrated to the national context and culture informing the country's learning health system hosting the embedded scholar. Thus, the proposed general orders are not all‐inclusive, nor serve as a “one size fits all” template. Indeed, the World Health Organization (WHO) suggests between and within‐country variance regarding readiness to utilize embedded researchers, and integrate the evidence they generate.^69^ Successes evidenced by embedded researchers in Chad, Nigeria, and India^70^ may subsequently encourage care systems to embrace other embedded researchers. These proposed general orders potentially provide a baseline from which to consider whether/how embedded researchers' engagement practice and duties vary within and between countries. Variation notwithstanding, the general orders potentially provide some fundamental, foundational truths to advance evidence‐based scholarship across diverse settings. For example, Varallyay and colleagues,^71^ observing embedded research in Latin America and the Caribbean, noted [t]eams able to maintain a focus on a common purpose fostered productive interactions among members despite differing viewpoints, in some circumstances contributing to pursuit of multiple complimentary strategies to promote evidence use (p.ii107). These general orders may direct embedded researchers toward articulating ideals serving a common purpose. Future work may consider potential international variation regarding whether/how embedded researchers assume and operationalize the proposed general orders.

## 2 SUMMARY

This discussion introduces learning health system stakeholders to a set of tenets—general orders—that may inform and guide embedded researchers in developing evidence to inform clinical practice; these general orders intend to both inform embedded researchers' identity, and encourage team‐based research equitably benefiting the patients, providers, and communities learning health systems serve. The general orders outlined above may remind embedded researchers, their partners, and their parent organizations that embedded researcher's identity, not merely their skills, represents the ultimate “value‐added” to learning health systems. Brandt and Gardner^72^ caution that “if collegiality is accomplished by capitulation to a reductionist biomedical paradigm in which public health primarily comes to help evaluate the safety, efficacy, quality and costs of biomedical interventions, something central will be lost: a powerful research and practical agenda concerning the social, cultural, and economic determinants of disease and suffering” (p.713). Likewise, embedded scholars viewed simply as instruments of model application and analysis may serve only to improve fundamentally flawed infrastructures. The general orders imbue embedded researchers with an ethical identity positioning them to re‐imagine learning health systems in a manner advancing the egalitarian application of the scientific method. Insights gleaned may evolve learning health systems toward both care equity and efficiency. These aspirations may position learning health systems to assuage the social, cultural, and economic determinants of disease Brandt and Gardner observe. Hopefully, the discourse encourages reflection, debate, and engagement, particularly among early career investigators embarking on their embedded researcher journey.

## CONFLICT OF INTEREST

The author affirms he has no conflict of interest to declare.

## References

[1] Greene SM , Reid RJ , Larson EB . Implementing the learning health system: from concept to action. Ann Intern Med. 2012;157(3):207‐210. 22868839 10.7326/0003-4819-157-3-201208070-00012

[2] Gould MK , Sharp AL , Nguyen HQ , et al. Embedded research in the learning healthcare system: ongoing challenges and recommendations for researchers, clinicians, and health system leaders. J Gen Intern Med. 2020. 10.1007/s11606-020-05865-4 PMC7728937 32472492

[3] Vindrola‐Padros C , Pape T , Utley M , Fulop NJ . The role of embedded research in quality improvement: a narrative review. BMJ Qual Saf. 2017;26(1):70‐80. 10.1136/bmjqs-2015-004877 PMC5256405 27129492

[4] Government of Canada‐Canada Institutes of Health Research : Embedded clinician researcher salary awards. https://cihr-irsc.gc.ca/e/50368.html. Accessed November 23, 2020.

[5] Government of Canada‐Canadian Institutes of Health Research : The health system impact fellowship. https://cihr-irsc.gc.ca/e/51211.html. Accessed November 23, 2020.

[6] Canadian Health Services and Policy Research Alliance : Report from the working group on training, December 7, 2015. http://ihpme.utoronto.ca/wp-content/uploads/2014/12/CHSPR-Alliance_Final_Dec7.pdf. Accessed November 23, 2020.

[7] Forrest CB , Chesley FD Jr , Tregear ML , Mistry KB . Development of the learning health system researcher Core competencies. Health Serv Res. 2018;53(4):2615‐2632. 28777456 10.1111/1475-6773.12751 PMC6051975

[8] Rizo CA , Jadad AR , Enkin M . What's a good doctor and how do you make one? Doctors should be good companions for people. BMJ (Clin Res Ed). 2002;325(7366):711‐711. PMC1124230 12351369

[9] Shaw Q . On aphorisms. Br J Gen Pract. 2009;59(569):954‐955.

[10] Kumar A , Allaudeen N . To cure sometimes, to relieve often, to comfort always. JAMA Intern Med. 2016;176(6):731‐732. 27110667 10.1001/jamainternmed.2016.1220

[11] Holmes CL , Harris IB , Schwartz AJ , Regehr G . Harnessing the hidden curriculum: a four‐step approach to developing and reinforcing reflective competencies in medical clinical clerkship. Adv Health Sci Educ Theory Pract. 2015;20(5):1355‐1370. 25319835 10.1007/s10459-014-9558-9

[12] Stokols D , Hall KL , Taylor BK , Moser RP . The science of team science: overview of the field and introduction to the supplement. Am J Prev Med. 2008;35(2 Suppl):S77‐S89. 18619407 10.1016/j.amepre.2008.05.002

[13] Haselager P , Mecacci G . Superethics instead of superintelligence: know thyself, and apply science accordingly. AJOB Neurosci. 2020;11(2):113‐119. 32228384 10.1080/21507740.2020.1740353

[14] Touati N , Rodríguez C , Paquette M‐A , Maillet L , Denis J‐L . Professional role identity: at the heart of medical collaboration across organisational boundaries. Int J Integr Care. 2019;19(2):1‐1. 10.5334/ijic.4184 PMC6450244 30971867

[15] Michalska‐Smith M . Describing a culture from within. AMA J Ethics. 2015;17(2):108‐110. 25676221 10.1001/virtualmentor.2015.17.02.fred2-1502

[16] Pijnenburg MAM , Gordijn B , Vosman FJH , HHAMJ T . Catholic healthcare organizations and the articulation of their identity. HEC Forum. 2008;20(1):75‐97. 18425587 10.1007/s10730-008-9063-8 PMC2797850

[17] Mannion R , Davies H . Understanding organisational culture for healthcare quality improvement. BMJ. 2018;363:k4907. 30487286 10.1136/bmj.k4907 PMC6260242

[18] Shanafelt TD , Schein E , Minor LB , Trockel M , Schein P , Kirch D . Healing the professional culture of medicine. Mayo Clin Proc. 2019;94(8):1556‐1566. 31303431 10.1016/j.mayocp.2019.03.026

[19] Gusfield J , Riesman D . Faculty culture and academic careers: some sources of innovation in higher education. Sociol Educ. 1964;37:281‐305.

[20] Gaff JG , Wilson RC . Faculty cultures and interdisciplinary studies. J High Educ. 1971;42:186‐201.

[21] Braxton JM . Norms and the work of colleges and universities: introduction to the special issue—norms in academia. J High Educ. 2010;81(3):243‐250.

[22] Dawson DL , Meadows KN , Kustra E , Hansen KD . Perceptions of institutional teaching culture by tenured, tenure‐track, and sessional faculty. Can J High Educ. 2019;49(3):115‐128.

[23] Lyken‐Segosebe DE , Braxton JM , Hutchens MK , Harris E . Codes of conduct for undergraduate teaching in four types of colleges and universities. Innov High Educ. 2018;43(4):289‐302.

[24] Daniels N . Understanding physician power: a review of the social transformation of American medicine. Philos Publ Aff. 1984;13(4):347‐357. 11654886

[25] Tilburt JC . Addressing dual agency: getting specific about the expectations of professionalism. Am J Bioeth AJOB. 2014;14(9):29‐36. 10.1080/15265161.2014.935878 25127273

[26] Bamford R . Getting even more specific about physicians' obligations: justice, responsibility, and professionalism. Am J Bioeth. 2014;14(9):46‐47. 10.1080/15265161.2014.935891 25127279

[27] Tilburt JC . Response to open peer commentaries on "Addressing dual agency: getting specific about the expectations of professionalism". Am J Bioeth AJOB. 2014;14(10):W4‐W5. 25229600 10.1080/15265161.2014.947826

[28] Pannucci CJ , Wilkins EG . Identifying and avoiding bias in research. Plast Reconstr Surg. 2010;126(2):619‐625. 20679844 10.1097/PRS.0b013e3181de24bc PMC2917255

[29] Selby JV , Beal AC , Frank L . The Patient‐Centered Outcomes Research Institute (PCORI) national priorities for research and initial research agenda. JAMA. 2012;307(15):1583‐1584. 22511682 10.1001/jama.2012.500

[30] Kirkham JJ , Dwan KM , Altman DG , et al. The impact of outcome reporting bias in randomised controlled trials on a cohort of systematic reviews. BMJ. 2010;340:c365. 20156912 10.1136/bmj.c365

[31] Norris S , Holmer H , Ogden L , et al. Selective Outcome Reporting as a Source of Bias in Reviews of Comparative Effectiveness‐AHRQ Publication No. 12‐EHC110‐EF. Rockville, MD: Agency for Healthcare Research and Quality; 2012. 22993870

[32] Freedman DH . Lies, Damned Lies, and Medical Science John Ioannidis Has Proved That Much of What Gets Published in Medical Journals is Wrong: Does Your Doctor Know? . The Atlantic Monthly; 2010:76.

[33] Bastani H , Goh J , Bayati M . Evidence of upcoding in pay‐for‐performance programs. Manag Sci. 2019;65(3):1042‐1060.

[34] Waddell C . So much research evidence, so little dissemination and uptake: mixing the useful with the pleasing. Evid Based Nurs. 2002;5(2):38‐40. 10.1136/ebmh.4.1.3 11467070

[35] Brownson RC , Eyler AA , Harris JK , Moore JB , Tabak RG . Getting the word out: new approaches for disseminating public health science. J Public Health Manag Pract. 2018;24(2):102‐111. 28885319 10.1097/PHH.0000000000000673 PMC5794246

[36] Edwards DJ . Dissemination of research results: on the path to practice change. Can J Hosp Pharm. 2015;68(6):465‐469. 26715783 10.4212/cjhp.v68i6.1503 PMC4690672

[37] Ross‐Hellauer T , Tennant J , Banelyte V , Gorogh E , Luzi D . Ten simple rules for innovative dissemination of research. PLoS Comput Biol. 2020;16(4). 10.1371/journal.pcbi.1007704 PMC7161944 32298255

[38] Erves JC , Mayo‐Gamble TL , Malin‐Fair A , et al. Needs, priorities, and recommendations for engaging underrepresented populations in clinical research: a community perspective. J Community Health. 2017;42(3):472‐480. 27812847 10.1007/s10900-016-0279-2 PMC5408035

[39] Institute of Medicine Committee on U, Eliminating R, Ethnic Disparities in Health C . In: Smedley BD , Stith AY , Nelson AR , eds. Unequal Treatment: Confronting Racial and Ethnic Disparities in Health Care. Washington, DC: National Academies Press (US) Copyright 2002 by the National Academy of Sciences. . All rights reserved; 2003. 25032386

[40] Thomas SB , Casper E . The burdens of race and history on black people's health 400 years after Jamestown. Am J Public Health. 2019;109(10):1346‐1347. 31483719 10.2105/AJPH.2019.305290 PMC6727285

[41] Barr‐Walker J , Bass MB , Werner DA , Kellermeyer L . Measuring impostor phenomenon among health sciences librarians. J Med Libr Assoc. 2019;107(3):323‐332. 31258438 10.5195/jmla.2019.644 PMC6579590

[42] Ward EC , Hargrave C , Brown E , Halkett G , Hogg P . Achieving success in clinically based research: the importance of mentoring. J Med Radiat Sci. 2017;64(4):315‐320. 28653426 10.1002/jmrs.234 PMC5715317

[43] Tai JHM , Canny BJ , Haines TP , Molloy EK . Implementing peer learning in clinical education: a framework to address challenges in the “real world”. Teach Learn Med. 2017;29(2):162‐172. 27997224 10.1080/10401334.2016.1247000

[44] Thorpe KE , Zwarenstein M , Oxman AD , et al. A pragmatic‐explanatory continuum indicator summary (PRECIS): a tool to help trial designers. J Clin Epidemiol. 2009;62(5):464‐475. 19348971 10.1016/j.jclinepi.2008.12.011

[45] Riddick FA Jr . The code of medical ethics of the american medical association. Ochsner J. 2003;5(2):6‐10. PMC3399321 22826677

[46] American Public Health Association : Public health code of ethics. https://www.apha.org/-/media/files/pdf/membergroups/ethics/code_of_ethics.ashx. 10.2190/NS.20.3.l 20943481

[47] Choo E , Moyer D . Sexual harassment between health care workers and safety culture. In: Commission TJ , ed. High Reliabiity Healthcare; 2020.

[48] Guilfoyle J , Kelly L , St Pierre‐Hansen N . Prejudice in medicine: our role in creating health care disparities. Can Fam Phys. 2008;54(11):1511‐1520. PMC2592307 19005106

[49] Serafini K , Coyer C , Brown Speights J , et al. Racism as experienced by physicians of color in the health care setting. Fam Med. 10.22454/FamMed.2020.384384 32267524

[50] Evans MK , Rosenbaum L , Malina D , Morrissey S , Rubin EJ . Diagnosing and treating systemic racism. N Engl J Med. 2020;383(3):274‐276. 32521155 10.1056/NEJMe2021693 PMC11701713

[51] Williams DR , Mohammed SA . Racism and health I: pathways and scientific evidence. Am Behav Sci. 2013;57(8). 10.1177/0002764213487340. PMC3863357 24347666

[52] Chew BH , Lee PY , Ismail IZ . "Personal mission statement": an analysis of medical students' and general practitioners' reflections on personal beliefs, values and goals in life. Malays Fam Phys. 2014;9(2):26‐33. PMC4399405 25893068

[53] Ramia E , Salameh P , Btaiche IF , Saad AH . Mapping and assessment of personal and professional development skills in a pharmacy curriculum. BMC Med Educ. 2016;16:19. 26772809 10.1186/s12909-016-0533-4 PMC4715283

[54] Motley RJ , McMullin A . Developing your professional career plan. Fam Pract Manag. 2020;27(4):21‐24. 32660228

[55] Warden GL , Institute of M . Redesigning Continuing Education in the Health Professions. Washington, DC: National Academies Press; 2010. 25032352

[56] Rosen MA , DiazGranados D , Dietz AS , et al. Teamwork in healthcare: key discoveries enabling safer, high‐quality care. Am Psychol. 2018;73(4):433‐450. 29792459 10.1037/amp0000298 PMC6361117

[57] Mitchell PH , Institute of M, Institute of M, Roundtable on V, Science‐Driven Health C, Best Practices Innovation C Core principles & values of effective team‐based health care (Discussion Paper). 2012; https://nam.edu/wp-content/uploads/2015/06/VSRT-Team-Based-Care-Principles-Values.pdf.

[58] Menear M , Blanchette M‐A , Demers‐Payette O , Roy D . A framework for value‐creating learning health systems. Health Res Policy Syst. 2019;17(1):79. 31399114 10.1186/s12961-019-0477-3 PMC6688264

[59] Harrison KL , Taylor HA . Organizational values in the provision of access to care for the uninsured. AJOB Empir Bioeth. 2016;7(4):240‐250. 28781981 10.1080/23294515.2016.1170075 PMC5540336

[60] Faden RR , Kass NE , Goodman SN , Pronovost P , Tunis S , Beauchamp TL . An ethics framework for a learning health care system: a departure from traditional research ethics and clinical ethics. Hastings Cent Rep. 2013; Spec No:S16‐S27. 10.1002/hast.134 23315888

[61] Reiser SJ . The ethical life of health care organizations. Hastings Cent Rep. 1994;24(6):28‐35. 7860286

[62] Winkler EC , Gruen RL . First principles: substantive ethics for healthcare organizations. J Healthc Manag. 2005;50(2):109‐119. 15839325

[63] Ghaffar A , Langlois EV , Rasanathan K , Peterson S , Adedokun L , Tran NT . Strengthening health systems through embedded research. Bull World Health Organ. 2017;95(2):87. 28250505 10.2471/BLT.16.189126 PMC5327943

[64] Glasgow RE . Implementation science: methods models and opportunities to integrate evidence, policy and practice http://www.ucdenver.edu/academics/colleges/medicalschool/programs/crisp/training/Documents/CRISP%20Seminar%20Series%20Presentation_092612.pdf, 2018.

[65] Aarons GA , Hurlburt M , Horwitz SM . Advancing a conceptual model of evidence‐based practice implementation in public service sectors. Adm Policy Ment Health Ment Health Serv Res. 2011;38(1):4‐23. 10.1007/s10488-010-0327-7 PMC3025110 21197565

[66] Eccles MP , Armstrong D , Baker R , et al. An implementation research agenda. Impl Sci. 2009;4:18. 10.1186/1748-5908-4-18 PMC2671479 19351400

[67] Graber DR , Kilpatrick AO . Establishing values‐based leadership and value systems in healthcare organizations. J Health Hum Serv Adm. 2008;31(2):179‐197. 18998522

[68] Martin D , Miller AP , Quesnel‐Vallée A , Caron NR , Bilkis V , Marchildon GP . Canada's universal health‐care system: achieving its potential. Lancet. 2018;391(10131):1718‐1735. 29483027 10.1016/S0140-6736(18)30181-8 PMC7138369

[69] World Health Organization‐Changing Mindsets : Strategy on health policy and systems research. https://www.who.int/alliance-hpsr/alliancehpsr_changingmindsets_strategyhpsr.pdf?ua=1.

[70] Alliance for Health Policy and Systems Research‐Embedded Research : An innovative approach to improving immunization rates. 2018; https://www.who.int/alliance-hpsr/news/2018/embedded-hpsr/en/. Accessed Nov 17, 2020.

[71] Varallyay NI , Bennett SC , Kennedy C , Ghaffar A , Peters DH . How does embedded implementation research work? Examining core features through qualitative case studies in Latin America and the Caribbean. Health Policy Plann. 2020;35(Supplement_2):ii98‐ii111. 10.1093/heapol/czaa126 PMC7646734 33156937

[72] Brandt AM , Gardner M . Antagonism and accommodation: interpreting the relationship between public health and medicine in the United States during the 20th century. Am J Public Health. 2000;90(5):707‐715. 10800418 10.2105/ajph.90.5.707 PMC1446218

